# Fluorouracil Supplemented With Oxaliplatin or Irinotecan for Solid Tumors: Indications From Clinical Characteristics and Health Outcomes of Patients

**DOI:** 10.3389/fonc.2020.01542

**Published:** 2020-09-02

**Authors:** Zhao Qingwei, Hong Dongsheng, Lv Duo, Wang Youlei, Yu Songxia, Ye Ziqi, Li Lanjuan

**Affiliations:** ^1^Key Laboratory for Drug Evaluation and Clinical Research of Zhejiang Province, The First Affiliated Hospital, Zhejiang University School of Medicine, Hangzhou, China; ^2^State Key Laboratory for Diagnosis and Treatment of Infectious Diseases, National Clinical Research Centre for Infectious Diseases, Collaborative Innovation Centre for Diagnosis and Treatment of Infectious Diseases, College of Medicine, The First Affiliated Hospital, Zhejiang University, Hangzhou, China

**Keywords:** solid tumors, fluorouracil, FOLFIRI, FOLFOX, real-world research

## Abstract

Fluorouracil combined with oxaliplatin (FOLFOX) and fluorouracil combined with irinotecan (FOLFIRI) are both first-line clinical chemotherapy regimens. However, clinicians' selection of FOLFIRI or FOLFOX medication regimens and their effects on patients' health outcomes are not clear. The aim of this study was to evaluate the impacts on patient characteristics of FOLFIRI or FOLFOX medication regimen selection and the effects of each regimen on patients' health outcomes in a real-world setting. Three thousand seven hundred and twenty-five patients were retrieved and 610 of them were eventually included in this study based on the inclusion and exclusion criteria. The percentages of the TNM stage, cetuximab, bevacizumab, and tumor metastases between the FOLFIRI and FOLFOX groups were different (*P* < 0.001). In the multivariate Cox proportional hazards model, a significantly higher non-convalescent incidence of the FOLFOX group was found as compared with the FOLFIRI group (HR = 2.211, 95% CI = 1.257–3.888, *P* = 0.006). In conclusion, the TNM stage, whether combined with cetuximab or bevacizumab, and whether there was tumor metastasis presented as the key factors affecting medication selection between the FOLFIRI and FOLFOX regimens. The FOLFIRI regimen exhibited better effects on patients' long-term health outcomes than did the FOLFOX regimen. This study was registered on the World Health Organization International Clinical Trials Registry Platform (ChiCTR2000029201).

**Trial registration:** ChiCTR2000029201.

## Background

Cancer is a globally recognized major public health problem and one of the common diseases that seriously threaten human health (1). The current treatment options of cancer, such as comprehensive treatment, radiotherapy, chemotherapy, targeted therapy, immunotherapy, and other methods, have greatly improved the survival of cancer patients (2, 3). Among these treatment methods, chemotherapy plays a very important role because of its universality and effectiveness (4). Although chemotherapy is very effective, its relatively poor selectivity inevitably results in damage to normal cells, especially in the proliferative phase, thereby causing various adverse reactions (5). Moreover, considering the potential adverse effects of chemotherapy on patients' long-term quality of life, it is necessary to optimize the drug selection in tumor chemotherapy regimens in order to benefit patients' prognosis.

Fluorouracil is one of the important chemotherapy drugs for treating solid tumors such as digestive tract tumors, breast cancer, ovarian cancer, lung cancer, cervical cancer, and bladder cancer (6). Fluorouracil has often been used in combination with oxaliplatin or irinotecan for patients with solid tumors like colorectal cancer and gastric cancer (7). The combination of two drugs usually has a certain synergistic effect. In the latest National Comprehensive Cancer Network (NCCN) guidelines on the first-line palliative chemotherapy for advanced colorectal cancer, the recommended grades in fluorouracil combined with irinotecan (FOLFIRI) or fluorouracil combined with oxaliplatin (mFOLFOX6) are class I recommendations, meaning that the clinical use of both options is preferred (8). The MAVERICC Study compared the efficacy of FOLFIRI plus bevacizumab (FOLFIRI-BV) with mFOLFOX6 plus bevacizumab (mFOLFOX6-BV) in metastatic colorectal cancer patients, and the results showed that these two treatment groups had a similar progression-free survival (PFS) [hazard ratio (HR) = 0.79, 95% CI = 0.61–1.01, *P* = 0.06] and overall survival (OS) (HR = 0.76, 95% CI = 0.56–1.04, *P* = 0.09) (9). The GERCOR Study investigated two sequences in colorectal cancer patients: FOLFIRI followed by FOLFOX6 (group A) and FOLFOX6 followed by FOLFIRI (group B). The results demonstrated that the median survival was similar between these two groups (21.5 vs. 20.6 months, *P* = 0.99). In first-line therapy, the median PFS of group A had no significant difference compared to group B (8.5 vs. 8.0 months, *P* = 0.26) (10). Although there may be no significant difference in efficacy between the two regimens, it has been known that each chemotherapeutic drug has its own specific adverse effects (11). For example, oxaliplatin, a platinum complex anticancer drug, has a relatively small renal toxicity, while its neurotoxicity is dose-limiting (12). Irinotecan, a semi-synthetic derivative of camptothecin, has adverse reactions such as acute cholinergic syndrome (13).

Given the varying differences in adverse reactions between oxaliplatin and irinotecan, although there is no significant difference between the short-term efficacies of these two different combined chemotherapy regimens, it is assumed that there might be differences between their effects on the long-term survival or health outcomes of patients. Health outcome is a very broad concept, which is subjective, multidimensional, and holistic, and is generally used to comprehensively reflect patients' feelings about themselves in the real world (14, 15). In judging the curative efficacy, prolonged survival does not always mean improved health outcomes (16). Nowadays, the importance of health outcomes, equivalent to the remission rate and survival time, is increasingly attracting attention and has been regarded as an outcome indicator in cancer research (17, 18). In order to understand the impact of the FOLFIRI and mFOLFOX6 regimens on the health outcomes of cancer patients, in this study, these two chemotherapy regimens were evaluated and compared, for the first time to our best knowledge, among different solid tumor patients after adjusting the impact of relevant factors such as disease. The results of this comparison study will be useful in providing references for the clinical selection of chemotherapy regimens. After all, under the circumstances that the curative effect is considered to be of great importance, the patients' health outcomes may become an essential evaluation index for future clinical research.

## Data and Research

### Study Design

This is a bidirectional study based on retrospective data, specifically for the retrospective collection of solid tumor patients treated with FOLFIRI or mFOLFOX6 in our hospital from January 2016 to December 2018. This study was conducted in accordance with the Helsinki Declaration and has been approved by the hospital clinical committee (19). This study will follow the statement of Strengthening the Reporting of Observational Studies in Epidemiology (STROBE), and The STROBE Statement Checklist of cohort studies is shown in online Supplementary Appendix 1 (20).

### Study Participants

The individuals involved in this study are “patients diagnosed with solid tumors for the first time and using FOLFIRI or mFOLFOX6” who were treated at the First Affiliated Hospital of Zhejiang University Medical College from January 2016 to December 2018. The detailed inclusion criteria were as follows: (1) aged 18–75 years, male or female; (2) expected survival time was longer than 3 months; and (3) administration of FOLFOX or FOLFIRI chemotherapy regimen for the first time. The exclusion criteria included: (1) pregnant or lactating women; (2) heart, lung, kidney, and other important organ failures; (3) those who concurrently used irinotecan or oxaliplatin chemotherapy regimen; and (4) those who failed to perform tumor staging.

### Data Collection

The data collection was carried out independently by two researchers and then checked by a third researcher. The electronic medical record system was used for retrieving and confirming the data. The demographic and clinical data collected in this study included: patient medical record number (unique identification code), gender, age, height, weight, whether the patient was diagnosed with a solid tumor for the first time, whether FOLFIRI or mFOLFOX6 was used, the main diagnosis, other diagnosis, surgery history, history of lung metastases, bone marrow metastases, liver metastases, hypertension, diabetes, cetuximab or rituximab, admission and discharge time, and the discharge outcomes.

### Sample Size

By assuming a hazard ratio of 2 (mFOLFOX6 *vs*. FOLFIRI) to be of clinical importance (HR = 2), it was further assumed that about 80% of non-convalescent incidence (*d* = 0.8) may be observed during the research period. When *n* = *n*1 = *n*2 (*p*1 = *p*2 = 0.5), the sample size per treatment group needed to achieve a power of 80% at the level of significance (α = 0.05) was given by.

$$\begin{array}{l} \text{n}=\frac{{\left(z_{\alpha /2}+z_{\beta }\right)}^{2}}{log^{2}\left(\text{HR}\right)p1p2d}=\frac{{\left(1.96+0.84\right)}^{2}}{log^{2}\left(2\right)\times 0.5\times 0.5\times 0.8}\approx 82 \end{array}$$

### Short-Term Outcome Indicators

The short-term outcome indicators were formulated in accordance with the “Case Classification Quality Management and Classification Standards” and were judged by an attending physician. The specific judgment criteria included: (1) cure: complete disappearance of symptoms, normal organ function, and wound healing. The main symptoms of chronic diseases disappear and function returns to normal; (2) improvement: the clinical symptoms and organ function have improved significantly; (3) unhealed: no change or aggravation of the symptoms or signs after hospitalization or organ function has no improvement or decrease; (4) other: refers to those who have been delivered normally, have been discharged from the hospital without delivery, have been discharged without disease, have been discharged without treatment, and have been discharged normally from a healthy person after an abortion or sterilization operation, as well as patients who have been discharged and transferred automatically without treatment after admission; and (5) death: clinical death of any inpatient (within 24 h) for any reason.

### Follow-Up and Indicators of Health Outcome

Patients were followed up during their regular visits to the hospital and also followed up by telephone by the hospital follow-up center. The recorded follow-up information included follow-up time, patient identification, and rehabilitation. The indicators of health outcomes included the patient's rehabilitation status, which was defined by medication taking, diet, stool, sleep and exercise, or comprehensive functional exercise.

### Statistical Analysis

Continuous variables were presented as the mean ± standard deviations (SD) or the medians with the minimum and maximum values. Student's *t*-tests were used for calculating continuous variables. The categorical variables were presented as frequencies (percentages) and assessed using a chi-square test. In order to explore the association of FOLFIRI and FOLFOX with non-convalescent incidence, Cox proportional hazards regression models were performed. Factors that showed a significant association (*P* < 0.10) after univariate Cox analysis were entered into the multivariate Cox analysis. A value of *P* < 0.05 was considered to be statistically significant. All of the statistical analyses were performed using SPSS19.0 for Windows (SPSS, Inc., Chicago, IL). The Kaplan–Meier survival curve was coded using R version 3.5.1 (R Core Team). A sample size of 98 patients provides at least 89.5% power for the non-convalescent event. The power calculations were performed using Cox regression. The non-convalescent event rate was 0.765 (23 convalescents and 75 non-convalescents). The hazard ratio was 2.221 in the FOLFOX group as compared with the FOLFIRI group.

## Results

### Demographic and Clinical Data of All Eligible Patients

**Figure 1:**
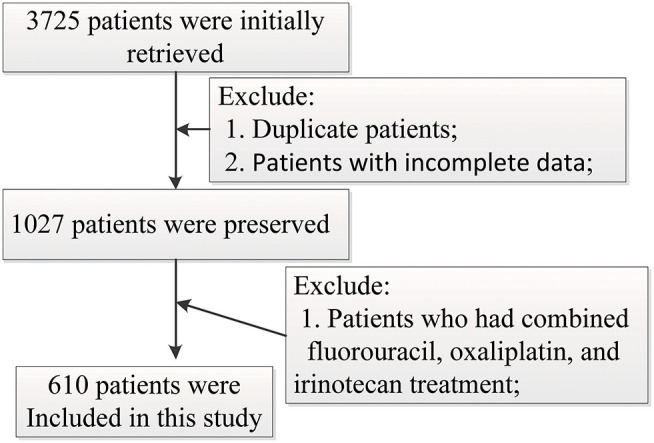
Patient screening process in this study.

Six hundred and ten gastrointestinal cancer patients were included in the study, of which 259 (42.5%) are colorectal cancer patients, 213 (34.9%) are colon cancer patients, 88 (14.4%) are stomach cancer patients, 13 (2.1%) are liver cancer patients, and 37 (6.1%) have other cancers. None of the patients chose other therapy in the later stage after FOLFOX or FOLFIRI in our study. One hundred and eighty-one (29.3%) patients were adopted in the FOLFIRI group and 429 (70.3%) were in the FOLFOX group. The patient screening process in this study is shown in Figure 1. Patients' characteristics are summarized in Table 1. The percentages of the TNM stages between the FOLFIRI group and the FOLFOX group were found to be statistically different (*P* < 0.001). The patients in stage IV tended to be treated with the FOLFIRI therapy. The percentage of patients using cetuximab or bevacizumab in the FOLFIRI group was statistically higher than that in the FOLFOX group (18.8% vs. 8.2% and 32.0% vs. 13.5%, respectively, both *P* < 0.001). Tumor metastases were also found to exhibit differences between the two groups (*P* < 0.001). In the FOLFIRI group, 59.1% of patients reported no metastases or non-liver or non-lung metastases, while the percentage in the FOLFOX group was 79.7%. No significant difference was found in age, gender, BMI, surgical operation, hypertension, and diabetes between the FOLFIRI group and the FOLFOX group (*P* > 0.05).

**Table 1:** Patient characteristics based on drug selection of FOLFIRI and FOLFOX.

	**Total**	**FOLFIRI**	**FOLFOX**	***P*-values**
*N* (%)	610	181 (29.7%)	429 (70.3%)	
Age (years)	58.49 ± 10.81	58.53 ± 10.79	58.44 ± 10.83	0.927^*^
Gender (male, %)	405 (66.4%)	118 (65.2%)	287 (66.9%)	0.684^#^
BMI	22.3 ± 3.2	22.6 ± 3.0	22.2 ± 3.3	0.102^*^
TNM stage (%)				<0.001
Stages I-II	54	6 (3.3%)	48 (11.2%)	
Stage III	195	25 (13.8%)	170 (39.6%)	
Stage IV	361	150 (82.9%)	211 (49.2%)	
Cetuximab (yes, %)	69	34 (18.8%)	35(8.2%)	<0.001^#^
Bevacizumab (yes, %)	116	58 (32.0%)	58 (13.5%)	<0.001^#^
Tumor metastases				<0.001^#^
Lung metastases	34	17 (9.4%)	17 (4.0%)	
Liver metastases	118	55 (30.4%)	63 (14.7%)	
Lung and liver metastases	9	2 (1.1%)	7 (1.6%)	
Non-lung + non-liver metastases	449	107 (59.1%)	342 (79.7%)	
Surgical operation (yes, %)	328	101 (55.8%)	227 (52.9%)	0.514^#^
Hypertension (yes, %)	137	38 (21.0%)	99 (23.1%)	0.573^#^
Diabetes (yes, %)	62	21 (11.6%)	41 (9.6%)	0.445^#^

*FOLFIRI, fluorouracil combined with irinotecan; FOLFOX, fluorouracil combined with oxaliplatin*.

* *P-values calculated by Student's t-test*.

# *P-values calculated by chi-square test*.

### Follow-Up of Patients and Health Outcomes

**Figure 2:**
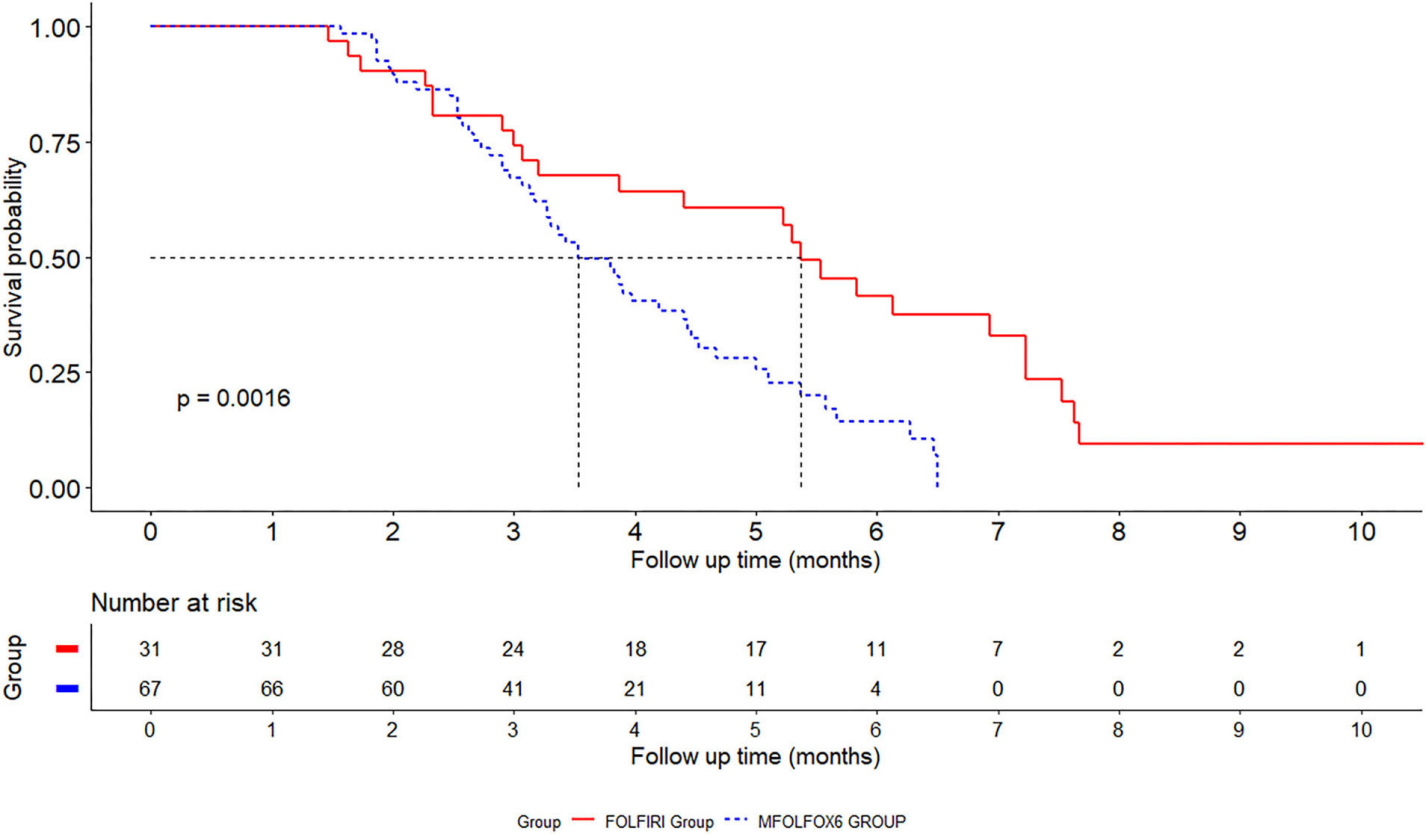
Kaplan–Meier survival curves for fluorouracil combined with oxaliplatin (FOLFOX) and fluorouracil combined with irinotecan (FOLFIRI) in gastrointestinal cancer patients.

One hundred and twelve patients were followed up (36 receiving FOLFIRI and 76 receiving FOLFOX) and their demographic and clinical data are listed in Table 2. Among the 112 patients, 14 (12.5%) were not followed prior to December 31, 2018. Ninety-eight patients were included for analyzing the prognosis of different therapy measures, of which 23 patients were in the convalescent phase and 75 patients were not convalescent. The median follow-up period was 3.6 months (ranging from 0.6 to 10.6 months). During the study period, seven (19.4%) patients were transferred to be convalescent in the FOLFIRI group and 16 (21.1%) in the FOLFOX group. The median convalescent times were 5.37 months in the FOLFIRI group and 3.53 months in the FOLFOX group. The patients were followed up at 3, 6, and 12 months. The convalescent ratios were 74.2, 41.7, and 9.38% in the FOLFIRI group and 67.2, 14.2, and 0% in the FOLFOX group. The FOLFOX group exhibited higher non-convalescent conditions as compared with the FOLFIRI group (log-rank test *P* = 0.0016; Figure 2).

**Table 2:** Characteristics of follow-up patients in the FOLFIRI and mFOLFOX6 groups.

	**Total**	**FOLFIRI**	**FOLFOX**	***P*-values**
*N*	112	36	76	
Age (years)	56.40 ± 10.72	53.94 ± 11.94	57.57 ± 9.96	0.095^*^
Gender (male, %)	76	24 (66.7%)	52 (68.4%)	0.853^#^
BMI	22.3 ± 3.3	22.7 ± 3.0	22.0 ± 3.4	0.330^*^
TNM stage (%)				0.705^#^
Stages I–II	6 (5.4%)	1 (2.8%)	5 (6.6%)	
Stage III	27 (24.1%)	9 (25.0%)	18 (23.7%)	
Stage IV	79 (70.5%)	26 (72.2%)	53 (69.7%)	
Cetuximab (yes, %)	19 (17.0%)	7 (19.4%)	12 (15.8%)	0.630^#^
Bevacizumab (yes, %)	35 (31.3%)	16 (44.4%)	19 (25.0%)	0.038^#^
Surgical operation (yes, %)	34 (30.4%)	9 (25.0%)	25 (32.9%)	0.396^#^
Hypertension (yes, %)	32 (28.6%)	10 (27.8%)	22 (28.9%)	0.898^#^
Diabetes (yes, %)	17 (15.2%)	3 (8.3%)	14 (18.4%)	0.165^#^
Tumor metastases				0.751^#^
Lung metastasis	10 (8.9%)	3 (8.3%)	7 (9.2%)	
Liver metastasis	27 (24.1%)	11 (30.6%)	16 (21.1%)	
Lung and liver metastases	7 (6.3%)	2 (5.6%)	5 (6.6%)	
Non-lung + non-liver metastases	68 (60.7%)	20 (55.6%)	48 (63.2%)	
Follow-up duration (months)	4.00 ± 1.77	4.95 ± 2.31	3.61 ± 1.30	<0.001^*^
Convalescent (*N*, %)	23 (20.5%)	7 (19.4%)	16 (21.1%)	0.888^#^

*FOLFIRI, fluorouracil combined with irinotecan; FOLFOX, fluorouracil combined with oxaliplatin*.

* *P-values calculated by Student's t-test*.

# *P-values calculated by chi-square test*.

Table 3 presents the baseline information of patients in the convalescent group and those in the non-convalescent group. The percentages of patients in the convalescent group receiving FOLFIRI and FOLFOX were 30.4% (7) and 69.6% (16), respectively, while those in the non-convalescent group were 24 (32.0%) and 51 (68.0%), respectively. No significant differences were found in age, gender, BMI, groups, TNM stage, cetuximab, bevacizumab, surgical operation, hypertension, diabetes, and tumor metastases between these two groups (*P* > 0.05).

**Table 3:** Characteristics of patients in the convalescent and non-convalescent groups.

	**Convalescent**	**Non-convalescent**	***P*-values**
*N*	23	75	
Age (years)	60.13 ± 7.97	55.85 ± 10.14	0.067^*^
Groups			0.888^#^
FOLFIRI	7 (30.4%)	24 (32.0%)	
FOLFOX	16 (69.6%)	51 (68.0%)	
Gender (male, %)	16 (69.6%)	53 (70.7%)	0.919^#^
BMI	22.3 ± 3.2	22.3 ± 2.9	0.981^*^
TNM stage (%)			0.737^#^
Stages I–II	1 (4.3%)	2 (2.7%)	
Stage III	7 (30.4%)	18 (24.0%)	
Stage IV	15 (65.2%)	55 (73.3%)	
Cetuximab (yes, %)	6 (26.1%)	11 (14.7%)	0.206^#^
Bevacizumab (yes, %)	9 (39.1%)	22 (29.3%)	0.377^#^
Tumor metastases			0.128^#^
Lung metastasis	0 (0%)	10 (13.3%)	
Liver metastasis	4 (17.4%)	20 (26.7%)	
Lung and liver metastases	1 (4.3%)	5 (6.7%)	
Non-lung + non-liver metastases	18 (78.3%)	40 (53.3%)	
Surgical operation (Yes, %)	6 (26.1%)	21 (28.0%)	0.857^#^
Hypertension (Yes, %)	9 (39.1%)	21 (28.0%)	0.311^#^
Diabetes (Yes, %)	5 (21.7%)	9 (12.0%)	0.243^#^

*FOLFIRI, fluorouracil combined with irinotecan; FOLFOX, fluorouracil combined with oxaliplatin*.

* *P-values calculated by Student's t-test*.

# *P-values calculated by chi-square test*.

The results of the above study indicated that the median improvement time in the FOLFIRI group was 5.3 months while that in the FOLFOX group was 3.8 months, and the overall improvement rate of the FOLFIRI group was higher than that of the FOLFOX group.

### Cox Proportional Hazards Regression Models

Tables 4, 5 show the HRs of non-convalescent incidence according to univariate and multivariate Cox proportional hazards models. In the univariate Cox proportional hazards model, the FOLFOX group was found to be significantly associated with a higher non-convalescent incidence as compared with the FOLFIRI group (HR = 2.404, 95% CI = 1.373–4.211, *P* = 0.002). Patients receiving cetuximab showed a lower non-convalescent incidence than those not receiving cetuximab (HR = 0.487, 95% CI = 0.252–0.942, *P* = 0.033). Furthermore, a multivariate Cox proportional hazards model was carried out to confirm the association. It was found that there was also a significantly higher non-convalescent incidence in the FOLFOX group as compared with the FOLFIRI group (HR = 2.211, 95% CI = 1.257–3.888, *P* = 0.006). However, a marginally significant association was found between the cetuximab and non-cetuximab groups (*P* = 0.091).

**Table 4:** Univariate Cox analysis of the long-term prognosis of cancer patients.

**Covariate**	**HR (95% CI)**	***P*-values**
Age (years)	1.002 (0.981–1.025)	0.836
Gender (Male, %)	1.068 (0.648–1.76)	0.797
FOLFOX vs. FOLFIRI	2.404 (1.373–4.211)	0.002
TNM stage (ref = stages I–II)	1.000	0.862
Stage III	0.838 (0.193–3.644)	0.814
Stage IV	0.970 (0.235–4.010)	0.967
Cetuximab	0.487 (0.252–0.942)	0.033
Bevacizumab	0.846 (0.514–1.393)	0.511
Surgical operation	0.882 (0.530–1.465)	0.627
Hypertension	1.059 (0.633–1.771)	0.827
Diabetes	1.228 (0.605–2.493)	0.569
Tumor metastases (ref = non-lung + non-liver metastases)	1.000	0.619
Lung metastasis	1.603 (0.797–3.226)	0.186
Liver metastasis	1.126 (0.657–1.928)	0.666
Lung and liver metastases	1.037 (0.403–2.668)	0.941

*FOLFIRI, fluorouracil combined with irinotecan; FOLFOX, fluorouracil combined with oxaliplatin*.

**Table 5:** Multivariate Cox analysis of long-term prognosis in cancer patients.

**Covariate**	**HR (95% CI)**	***P*-values**
FOLFOX *vs*. FOLFIRI	2.211 (1.257–3.888)	0.006
Cetuximab	0.561 (0.287–1.096)	0.091

*FOLFIRI, fluorouracil combined with irinotecan; FOLFOX, fluorouracil combined with oxaliplatin*.

The results of the above study indicate that patients receiving cetuximab showed a lower non-convalescent incidence than those not receiving cetuximab, and the TNM stage, whether cetuximab or bevacizumab was required, and whether tumor metastasis was present were the factors affecting the choice of FOLFIRI and FOLFOX drugs.

## Discussion

FOLFIRI and FOLFOX are first-line treatment options for gastrointestinal cancers, such as colorectal cancer and gastric cancer (8, 21). Although there is no significant difference in the efficacy of these two chemotherapy regimens, there might be discrepancies in their effects on the quality of life of patients. Real-world research has become an important direction for clinical research, and more and more real-world research evidence has become an essential source of evidence for clinical decision making (22). To the best of our knowledge, this study, for the first time, carried out a real-world research based on the effects of fluorouracil combined with oxaliplatin or irinotecan on the health outcomes of patients with solid tumors, thus providing a reference for the selection of clinical drug regimens.

All enrolled patients were with solid tumors and used FOLFIRI or FOLFOX for the first time. The main findings of this study include: (1) TNM staging, whether cetuximab or bevacizumab was required, and whether tumor metastasis was a factor affecting the choice of FOLFIRI and FOLFOX drugs. (2) The median improvement time in the FOLFIRI group was 5.3 months while for the FOLFOX group was 3.8 months. (3) The overall improvement rate of the FOLFIRI group was higher than that of the FOLFOX group, although the situation was reversed in the 2-month follow-up.

Although both the FOLFIRI and FOLFOX programs are clinical first-line medications, there are differences in the patient population using these two programs. FOLFOX programs are adopted more in TNM stages I–III, while FOLFIRI programs are used more in stage IV, indicating that TNM staging has a guiding significance in selecting FOLFIRI or FOLFOX regimens. In addition, for patients who need to take combined cetuximab or bevacizumab, the FOLFIRI regimen would be more appropriate, while for patients with lung and liver metastases, FOLFIRI would be a better choice. In summary, the results suggested that, in the real-world setting, for patients in TNM stage IV, with lung and liver metastases and clinical considerations requiring the combination of cetuximab or bevacizumab, the FOLFIRI regimen would be the preferred treatment program.

The effect of FOLFIRI on patients' long-term health outcomes appeared to be better than that of FOLFOX, although the condition for short-term quality of life was the opposite, presumably due to their difference in toxicity. Oxaliplatin's accumulative neurotoxicity and dose-limiting characteristics may lead to poor long-term quality of life for patients (23, 24). When the cumulative dose of oxaliplatin is up to 800 mg/m^2^, the risk of persistent symptoms will be up to 15%, and persistent neuropathy symptoms may worsen patients' quality of life (25). Irinotecan is a cycle-specific tumor treatment drug that acts in the S phase (26). It has toxicity on the gastrointestinal system and can induce early-onset diarrhea (27). Studies have shown that drug-related diarrhea that occurred 24 h after using irinotecan was dose-dependent, with incidence rates of 80–90%, among which grades 3–4 accounted for 39%, suggesting that the short-term effect of FOLFIRI was inferior to FOLFOX on patients' health outcomes (28, 29). It is the ultimate goal of treatment to prolong the overall survival and maintain the quality of life of patients in the advanced stage (30). At each step of treatment, special attention must be paid to the efficacy and safety in order to maintain maximum balance (30, 31). However, from the perspective of long-term health outcomes, the FOLFIRI program seems to be the best choice for patients with solid tumors.

It is necessary to mention that there are some deficiencies in this study which are worth considering for further research. Firstly, this was a single-center study, and some homogeneity might exist in the included cancer patients. Secondly, due to the limitations of follow-up, health outcomes were qualitatively evaluated on the basis of patients' medication taking, diet, defecation, sleep and exercise, or functional exercise rather than a systematic rating scale. Thirdly, different tumors may also have heterogeneity in the findings. Therefore, more randomized controlled trials (RCTs) and multicenter studies are still needed to confirm our findings in the future.

## Conclusion

Based on the actual situation in China, this study compared and evaluated the impact of patient characteristics on the selection between two widely used medication treatment regimens, i.e., FOLFIRI or FOLFOX regimen, as well as the effects of the regimen selection on patients' health outcomes in the real-world setting. The results of this study suggested that the TNM stage, whether combined with cetuximab or bevacizumab, and whether there was tumor metastasis were the key factors that affected the selection of these two regimens. However, more RCTs and multicenter studies will be necessary in future studies to confirm the above findings.

## Data Availability Statement

The raw data supporting the conclusions of this article will be made available by the authors, without undue reservation.

## Ethics Statement

The studies involving human participants were reviewed and approved by the ethics committee of the First Affiliated Hospital of College of Medicine of Zhejiang University in China. Written informed consent for participation was not required for this study in accordance with the national legislation and the institutional requirements.

## Author Contributions

ZQ and LL had the original idea for this article. HD and LD contributed to the study design and paper writing. WY, YS, and YZ had completed clinical data collection and patient follow-up and reviewed previous studies. All authors read and approved the final version of the article.

## Conflict of Interest

The authors declare that the research was conducted in the absence of any commercial or financial relationships that could be construed as a potential conflict of interest.
